# Evaluación de la eficacia biológica y de la sensibilidad de *Aedes aegypti* a los insecticidas piretroides deltametrina y ciflutrina durante el brote del virus Zika en Kuna Yala, Panamá

**DOI:** 10.7705/biomedica.6746

**Published:** 2023-06-30

**Authors:** Lorenzo Cáceres, Cipriano Ayarza, Damaris Bernal

**Affiliations:** 1 Departamento de Entomología Médica, Instituto Conmemorativo Gorgas de Estudios de la Salud, Ciudad de Panamá, Panamá Instituto Conmemorativo Gorgas de Estudios de la Salud Ciudad de Panamá Panamá; 2 Sección de Control de Vectores, Región de Salud, Kuna Yala, Panamá Región de Salud Kuna Yala Panamá

**Keywords:** Aedes, resistencia a los insecticidas, control de vectores de las enfermedades, dengue, virus Zika., Aedes, insecticide resistance, vector control of disease, dengue, Zika virus.

## Abstract

**Introducción.:**

El desarrollo de la resistencia a insecticidas de *Aedes aegypti* representa una gran amenaza para la salud pública. La vigilancia y el monitoreo de la eficacia biológica a los insecticidas y la sensibilidad de las poblaciones de *Aedes aegypti* es de fundamental importancia para prolongar la vida útil de estas moléculas.

**Objetivo.:**

Evaluar la eficacia biológica de los insecticidas deltametrina y ciflutrina y la sensibilidad de poblaciones de *Aedes aegypti* a estos insecticidas durante el brote epidémico de virus del Zika en Kuna Yala, Panamá.

**Métodos y materiales.:**

Se evaluó la eficacia biológica de la deltametrina y la ciflutrina, y la sensibilidad a estos insecticidas de poblaciones de la cepa *Aedes aegypti* Ustupo, mediante bioensayos estandarizados por la Organización Mundial de la Salud durante el brote epidémico de virus del Zika en Kuna Yala, Panamá.

**Resultados.:**

En los bioensayos con *Aedes aegypti* Ustupo se observó posible resistencia a deltametrina y a ciflutrina con un porcentaje de mortalidad del 95,3 y 94 %, respectivamente. Se registró baja eficacia biológica con la cepa *Aedes aegypti* Ustupo para la deltametrina y la ciflutrina, con medias de porcentajes de mortalidad de 75 y 31,1 %, en el intradomicilio, mientras que en el peridomicilio fue de 63,7 y 26,1 %, respectivamente.

**Conclusión.:**

Los resultados de este estudio representan un desafío que debe enfrentar el Programa Nacional de Control de Aedes para lograr cuidar y mantener el efecto tóxico de los insecticidas aplicados contra las poblaciones de *Aedes*. Es necesario que el Programa Nacional de Control de Aedes establezca unos lineamientos de manejo de la resistencia para caracterizarla y evaluar la distribución geográfica de las poblaciones afectadas. Lo anterior con el propósito de garantizar la sostenibilidad de las intervenciones antivectoriales contra las poblaciones de *Aedes*.

## Abstract (en)

**Introduction.:**

The development of resistance to insecticides in *Aedes* a*egypti* represents a major threat to public health. Surveillance and monitoring of the biological efficacy and sensibility of *Aedes aegypti* populations to insecticides is fundamental to prolong the useful life of insecticide molecules.

**Objective.:**

To evaluate the biological efficacy of deltamethrin and cyfluthrin and sensibility to insecticides in *Aedes aegypti* during the zika epidemic outbreak in Kuna Yala, Panama. Methods and materials. We assessed the biological efficacy of deltamethrin and cyfluthrin, and sensibility in the strain *Aedes aegypti* Ustupo using bioassays standardized by the World Health Organization during the Zika epidemic outbreak in Kuna Yala, Panama.

**Results.:**

In the bioassays with *Aedes aegypti* Ustupo, we observed a possible resistance to deltamethrin and cyfluthrin with a mortality rate of 95,3 and 94%, respectively. The obtained results registered low biological efficacy of deltamethrin and cyfluthrin with average percentages of mortality of 75 and 31.1% in the intradomicile, and 63,7 and 26.1% in the peridomicile.

**Conclusion.:**

The results of this study represent a challenge for the National *Aedes* Control Program to care for and maintain the toxic effect of insecticides applied against *Aedes* populations. There is a need for the National *Aedes* Control Program to establish some guidelines about resistance assessment and resistant populations’ geographic distribution to guarantee the sustainability of anti-vector interventions against *Aedes* populations.

Los virus del dengue (DENV) son transmitidos por dos especies de mosquitos, *Aedes aegypti* y *Aedes albopictus*, que se crían y distribuyen en ambientes urbanos. Por esta razón, el dengue es una enfermedad predominantemente urbana. Según estimaciones recientes, unos 390 millones de personas se infectan anualmente con los virus del dengue y 96 millones llegan a manifestar clínicamente la infección ^1^. El virus del chikungunya (CHIVK) y el virus del Zika (ZIKV), otros dos virus causantes de arbovirosis, son transmitidos por los mismos vectores y en las últimas décadas han tenido un gran impacto en los brotes epidémicos urbanos de las Américas, África y Polinesia ^2^.

Una epidemia generalizada de infección por ZIKV fue reportada en 2015 en Centroamérica, Suramérica y el Caribe ^3^. En mayo del 2015, la Organización Mundial de la Salud (OMS) registró la primera transmisión local de ZIKV en el continente americano y se identificaron casos autóctonos en Brasil ^4^^,^^5^. El ZIKV se ha confirmado con transmisión local en 51 países y territorios de América ^6^^,^^7^. En Panamá, desde la reemergencia del dengue en 1993 hasta el 2021, se han registrado 98.581 casos acumulados de dengue, 219 casos de dengue grave y 79 defunciones. Desde que emergió en el 2014, el chikungunya ha registrado 410 casos acumulados hasta el 2021, mientras que desde que se registraron los primeros casos de Zika en el 2015 hasta el 2021, se han registrado 1.371 casos acumulados ^8^.

En los últimos años ha ocurrido un incremento significativo de los casos de dengue en algunos países de las Américas, que unido a la introducción de ZIKV y CHIKV, han generado una situación epidemiológica más compleja y representan un desafío adicional para los sistemas de salud pública ^9^.

El principal vector de DENV en todo el mundo son los mosquitos *Aedes* aegypti. Esta especie se ha adaptado a las zonas urbanas, en las áreas del peridomicilio, se cría a menudo en recipientes artificiales y pica casi exclusivamente a los humanos ^10^^,^^11^. La circulación de la enfermedad va emparejada con la distribución geográfica de *Ae. aegypti*^12^. El control vectorial es el método principal disponible para controlar las infecciones transmitidas por vectores y tiene como objetivo limitar la transmisión de agente patógenos al reducir o eliminar el contacto hombre-vector ^13^. Este es el método utilizado para controlar el CHIKV y el ZIKV y proteger así a las poblaciones humanas. Para algunas enfermedades, como el dengue, existe una vacuna que está autorizada, pero no ampliamente utilizada debido a problemas de seguridad ^14^.

Algunos programas de control de *Ae. aegypti* se centran en la vigilancia y en la eliminación de los sitios de cría, y en campañas de saneamiento ambiental para minimizar los riesgos de proliferación del vector. No obstante, enfrentan desafíos operativos por la presencia de múltiples tipos de criaderos en los sitios donde se aplican estas estrategias.

Por esta razón, y ante la presencia de elevados índices de infestación de *Ae. aegypti* y riesgos potenciales de transmisión, se lleva a cabo una etapa intensiva basada fundamentalmente en el uso de insecticidas ^15^^,^^16^. Las aplicaciones de insecticidas en aerosol a volumen ultrabajo son utilizadas para controlar los mosquitos en estadio adulto cuando están en vuelo o en reposo. Durante más de 60 años, la aspersión de insecticidas en forma de aerosol ha sido una herramienta crucial para combatir los mosquitos. Dos tecnologías han dominado su aprovechamiento y uso continuo en todo el mundo: la nebulización térmica ^17^, y los aerosoles de niebla fría ^18^. De este modo, se ha logrado prevenir y controlar la transmisión viral. Sin embargo, la mayoría de las veces, quedan poblaciones sobrevivientes de *Ae. aegypti* que, junto con los riesgos ambientales y sociales, representan un peligro potencial para la ocurrencia de nuevos brotes o epidemias.

Los estudios llevados a cabo en numerosos lugares del mundo han demostrado la capacidad que tiene *Ae. aegypti* para desarrollar resistencia a los insecticidas, lo que se constituye en el principal problema que afecta las estrategias de los programas de control de vectores ^19^^,^^20^. La resistencia de *Ae. aegypti* a los insecticidas piretroides y organofosforados representa una amenaza creciente para la sostenibilidad de las estrategias de los programas de control vectorial ^21^^,^^22^. Desde la introducción de los insecticidas sintéticos en los programas sanitarios de las Américas, la presión selectiva ejercida contra poblaciones de *Ae. aegypti* ha generado el desarrollo de resistencia a varios de ellos, además de la aparición de resistencia cruzada y múltiple con otros insecticidas, lo cual reduce el número de alternativas efectivas y adecuadas para el control del vector ^16^^,^^23^.

En Panamá, desde el inicio del programa de control de vectores en 1956, las poblaciones de mosquitos transmisores de agentes patógenos han estado sometidas a una continua presión selectiva de insecticidas organoclorados, carbamatos, organofosforados y piretroides ^24^. El Programa Nacional de Control de Aedes del Ministerio de Salud, debido al registro constante de elevados índices de infestación, casos de dengue y la circulación de sus cuatro serotipos, y la emergencia de CHIKV y ZIKV en el país, ha mantenido el uso constante de los insecticidas piretroides deltametrina y ciflutrina para tratar de controlar la transmisión de estas entidades virales.

Durante el registro del primer brote epidémico de Zika en el país, ocurrido en la comarca de Kuna Yala en el 2015, con el registro de 38 de los 39 casos (97,4 %) de la enfermedad registrados a nivel nacional, la comunidad de Ustupo reportó 29 casos de Zika, el 74,4 % de los registrados a nivel nacional ^25^. En consideración a esta situación, el Gobierno Nacional declaró una “alerta sanitaria en todo el territorio nacional” y se declaró el territorio de la comarca Kuna Yala como “zona epidémica” sujeta a control sanitario, ante el riesgo de propagación de la enfermedad por tratarse de un evento de salud pública de importancia nacional e internacional ^26^.

La principal acción antivectorial que se llevó a cabo fue la aplicación de los insecticidas deltametrina y ciflutrina con equipo de nebulización térmica de volumen ultrabajo en áreas peridomésticas o perifocales de las casas donde se sospechaba o se habían reportado infecciones humanas. También, se realizaron intervenciones de control físico para reducir las fuentes de transmisión que consistieron en la eliminación de criaderos activos y potenciales con participación comunitaria, y en la ejecución de actividades educativas. El control químico larvario se realizó mediante la aplicación de diflubenzurón y temefós en depósitos de agua de uso doméstico ^25^.

El Programa Nacional de Control de *Aedes* ha manifestado la necesidad de determinar el estado actual de la sensibilidad y la eficacia biológica de los insecticidas aplicados contra las poblaciones de *Aedes* en las distintas regiones endémicas del país. Lo anterior con el propósito de generar nuevas estrategias para evitar o retrasar el desarrollo de la resistencia en las poblaciones de *Aedes*.

El objetivo de este estudio fue evaluar la eficacia biológica y la sensibilidad de poblaciones de *Ae. aegypti* de la región de Ustupo a los insecticidas piretroides deltametrina y ciflutrina durante el brote de Zika en la comarca indígena de Kuna Yala.

## Materiales y métodos

### Descripción del área de estudio

La comarca-división política o territorio asignado a una población indígena definida dentro de Panamá-de Kuna Yala comprende alrededor de 2.341 km^2^ de superficie continental; está conformada por bosques tropicales intervenidos y áreas costeras de tierras bajas utilizadas por la población indígena kuna para cultivar principalmente palma de coco, plátano, banana y yuca, entre otros. Esta región cuenta con un archipiélago constituido por cerca de 365 pequeñas islas de coral, cuya ecología ha sido fuertemente modificada por acciones antropogénicas ^27^^,^^28^.

Kuna Yala está subdividida políticamente en cuatro corregimientos con 49 comunidades oficialmente reconocidas, la mayoría ubicadas en islas cercanas a la costa continental. En el 2015, la población de Kuna Yala estaba estimada en unos 42.395 habitantes ^29^. Cada comunidad está regida por autoridades tradicionales representadas por un líder político y espiritual llamado “Sahila” y por el Congreso General Kuna ^30^.

En general, las comunidades mantienen un saneamiento básico, pero carecen de un suministro de agua entubada las 24 horas. Debido a esto, la población tiene que almacenar agua en diferentes tipos de depósitos que se convierten en criaderos de *Ae. aegypti*. La temperatura promedio mensual en la región es de 26 a 27 °C, con una humedad relativa entre el 78 y el 90 % y una precipitación anual que oscila entre los 1.600 y los 3.000 mm^3 (^^31^. Esta región tiene normalmente un patrón de lluvia unimodal con una temporada seca que inicia generalmente a mediados de diciembre y va hasta abril, y una temporada lluviosa que inicia a mediados de mayo y va hasta diciembre.

### Sitio seleccionado

El Programa Nacional de Control de Aedes, considerando la magnitud del problema de salud pública que representa el registro continuo de brotes de los virus dengue, Zika y chikungunya, y la reciente emergencia de Zika en la comarca de Kuna Yala, planteó la necesidad de determinar el comportamiento de la eficacia biológica de los insecticidas piretroides deltametrina y ciflutrina, utilizados en aplicaciones de volumen ultrabajo, contra poblaciones de Aedes en las principales regiones endémicas ^25^.

**Figura 1.**
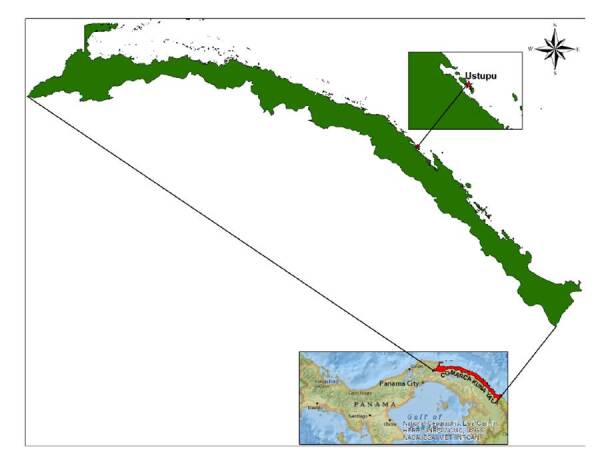
Localización geográfica de la comunidad de Ustupo en la comarca de Kuna Yala, Panamá * Comunidad seleccionada para el estudio

Para la evaluación de la sensibilidad y de la eficacia biológica de los insecticidas se seleccionó, en conjunto con el Ministerio de Salud de Panamá, la comunidad de Ustupo ubicada a los 9° 07’ 48.44” N y 77° 55’ 38.21” O, que cuenta con 120 viviendas y 2.300 habitantes (figura 1). Esta comunidad tiene reportes frecuentes de casos de dengue, altos índices de infestación, aplicaciones frecuentes de insecticidas y registro de la mayoría de los casos de zika en Kuna Yala ^25^.

### Recolecta de individuos de Aedes aegypti en Ustupo

Las muestras de *Ae. aegypti* de Ustupo en fases inmaduras (larvas y pupas) se recolectaron en criaderos activos localizados en el domicilio y en el peridomicilio de las viviendas entre las 08:00 y las 16:00 horas con ayuda del personal técnico del Programa Nacional de Control de Aedes. Todo el material biológico recolectado se colocó en envases especiales debidamente codificados y se transportó al Departamento de Entomología Médica del Instituto Conmemorativo Gorgas de Estudios de la Salud para su identificación, a nivel de especie, mediante el uso de claves taxonómicas de larvas de mosquitos ^32^.

### Cría y mantenimiento de cepas de mosquitos en condiciones de laboratorio

Una vez identificada la especie del material biológico recolectado en el campo, se obtuvieron los mosquitos adultos a partir de las larvas y las pupas de *Ae. aegypti* recolectadas en Ustupo. A estos se les denominó la generación F_0_. A partir de las posturas producidas de la F_0_ se obtuvo la primera generación (F_1_) de la cepa denominada “*Ae. aegypti* Ustupo” con la cual se levantó una colonia que fue criada y mantenida en condiciones controladas de laboratorio: con una temperatura promedio mínima de 28,5 °C (desviación estándar: DE = 0,5703) y máxima de 30,0 °C (DE = 0,0912), humedad relativa del 70 al 80% (DE = 0,3939) y con fotoperíodo de 12 horas día y 12 horas noche ^33^. El material biológico de la cepa *Ae. aegypti* Ustupo fue utilizado posteriormente para realizar los bioensayos de sensibilidad y pruebas de eficiencia biológica. Bajo las mismas condiciones se mantuvo la cepa de referencia sensible *Ae. aegypti* Rockefeller.

### Insecticidas utilizados

*Deltametrina: (s)-a-cyano-3-phenoxybenzyl cis-(IR)-3-(2,2-dibromovinyl)- 2,2-dimethyl cyclopropane carboxylate*. Tiene un 96,8 % de pureza y fue suministrada por Bayer, S. A. Central América, Costa Rica, y la presentación comercial K-Othrine^®^ CE 0,27 % w/v (Deltametrina), producto formulado en Guatemala por Bayer, S. A.

*Ciflutrina: (RS;SR)-alpha-cyano-4-fluoro-3- phenoxybenzyl (1 RS,3RS;1 RS,3SR) -3-(2,2-dichlorovinyl) -2,2-dimethylcyclopropanecarboxylate* del 98 % de pureza, suministrada por Bayer, S. A. Central América, Costa Rica y el formulado comercial Solfac^®^ UBV 1,5% w/v (Cyflutria), producto formulado en Guatemala por Bayer, S. A.

### Nebulización térmica con piretroides en el intradomicilio y el peridomiciiio

Como medida de control de calidad, las aplicaciones de las nebulizaciones térmicas de volumen ultrabajo de los insecticidas piretroides en el intradomicilio y en el peridomiciiio estuvieron a cargo de un mismo operario técnico del Programa Nacional de Control de Aedes de la comarca de Kuna Yala, con el fin de disminuir los sesgos inducidos por la manera de aplicar la técnica de nebulización dentro de las viviendas y fuera de ellas.

El equipo utilizado en las aplicaciones fue un nebulizador térmico portátil de volumen ultrabajo, marca Dyna-Fox Super Hawk II^®^, previamente calibrado de acuerdo con las normas de la OMS ^34^. El promedio de la temperatura y la humedad relativa durante los bioensayos fue de 27 ± 2 °C y del 70 al 80%m respectivamente. La velocidad del viento durante la aplicación fue de 5 a 8 km/h. Luego de terminar la jornada de aplicación, el equipo se recalibró para garantizar una aplicación adecuada en días subsiguientes.

Para la aplicación de la deltametrina se hizo una mezcla de un litro del formulado comercial K-Othrine CE 0,27% en cuatro litros de diésel (1:4) para una concentración final de 0,068 %. Por su parte, para la solución de ciflutrina se mezcló un litro del formulado comercial Solfac 1,5 % en 12 litros de diésel (1:12) para una concentración final de 0,125 % ^35^. La descarga para ambos insecticidas con el nebulizador térmico fue de 177 ml/min.

Se seleccionaron 60 viviendas para la aplicación de los insecticidas piretroides durante tres días consecutivos. La mayoría de las viviendas estaban construidas de acuerdo con el modelo de la cultura kuna (techo vegetal de hojas de palma, paredes de pequeños troncos de árboles colocados verticalmente, sin ventanas y piso de tierra) ^36^.

Para la selección de las casas se tuvo en consideración la accesibilidad y el permiso de los propietarios para poder ingresar y realizar los trabajos en las viviendas ^37^. Se seleccionaron seis sectores y diez viviendas por cada sector. Tres sectores fueron tratados con deltametrina durante tres días consecutivos y tres sectores con ciflutrina durante tres días consecutivos.

Las viviendas utilizadas como control estaban ubicadas a una distancia aproximada de 500 m de las viviendas tratadas. Las nebulizaciones térmicas de volumen ultrabajo se realizaron durante las primeras horas de la mañana (07:00 - 09:00), para evitar altas temperaturas y vientos variables, siguiendo la metodología de la OMS ^34^.

**Figura 2.**
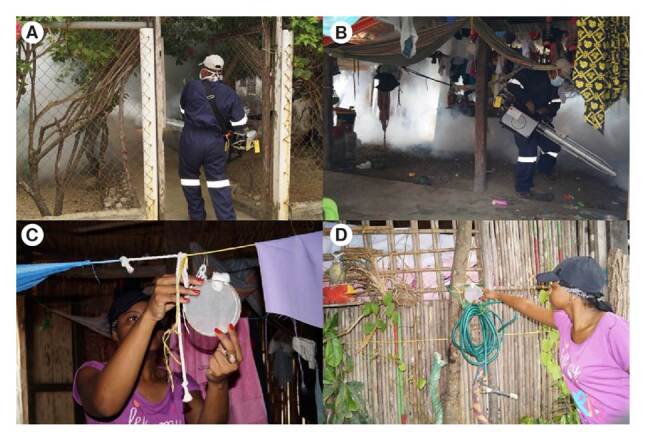
Comunidad de Ustupo, Kuna Yala, Panamá. Nebulización de las áreas. **A.** Peridomicilio. **B.** Intradomicilio. Colocación de las jaulas de exposición. **C.** Intradomicilio. **D.** Peridomicilio.

Antes de las aplicaciones de los insecticidas en el intradomicilio y el peridomicilio se solicitó a los dueños de las viviendas que cubrieran todos los alimentos, utensilios y agua, mantuvieran cerradas las ventanas y las puertas y, salieran de las casas por 30 minutos después de la nebulización térmica con el insecticida. El tiempo de aplicación de los insecticidas en el intradomicilio fue de cinco a diez segundos según el tamaño de la vivienda, mientras que el tiempo de aplicación promedio en el peridomicilio fue de 10 a 15 segundos, según el tamaño del peridomicilio (figura 2, imágenes A y B).

### Bioensayos de sensibilidad en mosquitos adultos Aedes aegypti

Las muestras de hembras adultas de la primera generación (F_1_ ) de *Ae. aegypti* Ustupo fueron expuestas a bioensayos de sensibilidad mediante papeles impregnados con los insecticidas piretroides deltametrina (0,1 %) y ciflutrina (0,1 %), usando la dosis diagnóstica y los tiempos de exposición establecidos por las normas estandarizadas de la OMS ^38^^,^^39^. Se utilizaron lotes de 25 hembras de dos a cuatro días de emergidas y alimentadas con sangre de equino preservada. Los bioensayos se hicieron una hora después de alimentar a los mosquitos.

La temperatura promedio registrada durante los bioensayos fue de 27 ± 2 °C y 70 ± 80% de humedad relativa. Cada prueba con los insecticidas evaluados contó con cuatro réplicas y sus respectivos controles. El tiempo de exposición fue de 60 minutos, durante los cuales se registró el número de mosquitos caídos a los 15, 30, 45 y 60 minutos.

Después del período de exposición, los mosquitos se trasladaron a las cámaras de recuperación y se colocó algodón humedecido en solución al 10 % de sacarosa como alimento durante el período de recuperación. El porcentaje de mortalidad se registró a las 24 horas. Se utilizó como patrón estándar de sensibilidad la cepa de referencia *Ae. aegypti* Rockefeller.

Se define como mosquitos resistentes a los insecticidas a aquellos que sobreviven 24 horas después de finalizar el bioensayo, y mosquitos sensibles, a los que murieron durante los 60 minutos siguientes al tiempo de exposición o dentro de las 24 horas del período de recuperación.

### Evaluación de la eficacia biológica de los insecticidas piretroides

En cada una de las 60 viviendas seleccionadas se realizaron aplicaciones para cada uno de los insecticidas (deltametrina y ciflutrina), durante tres días consecutivos.

En el estudio se colocaron un total de 360 jaulas codificadas, cada una con 15 hembras adultas de la cepa *Ae. aegypti* Ustupo (n=5.400). Se ubicaron tres jaulas en el intradomicilio y tres en el peridomicilio e igual cantidad de jaulas y mosquitos de la cepa sensible *Ae. aegypti* Rockefeller, colocadas a diferentes alturas (0,6 -1,2 m).

En el intradomicilio se colocó una jaula en el dormitorio, una en la sala y otra en la cocina. En el peridomicilio las tres jaulas se colocaron en los costados de las viviendas o lugares que se prestaban como sitios de reposo para los mosquitos que estuvieran a la sombra, frescos y húmedos (figura 2C y 2D).

Los mosquitos utilizados en este estudio tenían tres o cuatro días de emergidos. Las jaulas se colocaron en las viviendas 15 minutos antes de la aplicación del insecticida y las viviendas tratadas permanecieron cerradas durante 30 minutos después de la aplicación para evitar el escape de insecticida y lograr así el efecto deseado.

Posteriormente, se procedió a extraer las jaulas de las viviendas y se anotó el número de mosquitos caídos en cada una. Las jaulas con mosquitos fueron llevadas a la base y allí los mosquitos fueron trasladados a envases de reposo limpios, con algodón humedecido en solución al 10 % de sacarosa como alimento durante el período de recuperación. Los individuos se mantuvieron hasta las 24 horas en condiciones adecuadas de temperatura de 27 ± 2 °C y 70 - 80% de humedad relativa. El porcentaje de mortalidad de las muestras de *Ae. Aegypti*, expuestas a la aplicación de insecticidas piretroides, se registro 24 horas después.

### Análisis

La nebulización térmica con equipo de volumen ultrabajo se considera eficaz cuando la mortalidad de los mosquitos expuestos y la cobertura de aplicación del insecticida de las viviendas de un área son del 80 al 100 % ^40^.

Las poblaciones de mosquitos se clasificaron como resistentes o sensibles considerando los resultados de los bioensayos de adultos estandarizados por la OMS. Estos indican que indican que entre el 98 y el 100% de mortalidad es senstible; entre el 90 y el 97 % de mortalidad sugiere que puede estar desarrollándose resistencia, y menos del 90 % de mortalidad, indica resistencia ^38^. Se tuvo en cuenta la fórmula de Abbott, para corregir la mortalidad de los mosquitos expuestos a insecticidas, cuando esta osciló entre el 5 y el 20 % ^41^.

Los datos de porcentaje de mortalidad de *Ae. aegypti* Ustupo y *Ae. aegypti* Rockefeller, expuestos a deltametrina y ciflutrina, fueron analizados usando un modelo lineal binomial generalizado que consideró las siguientes variables: sitio de aplicación (intradomicilio o peridomicilio), exposición al insecticida (control, ciflutrina o deltametrina) y tiempo de exposición (1 y 24 horas).

### Consideraciones éticas

Antes de realizar el trabajo, se contactaron las autoridades tradicionales del Congreso Indígena de Ustupo para obtener el permiso para realizar el estudio.

Antes del inicio del trabajo de investigación se contó con el apoyo del personal técnico local de la etnia kuna, perteneciente al Programa Nacional de Control de Aedes. Ellos estaban acostumbrados a recibir a la población en sus casas durante las actividades de vigilancia y control del Aedes que se llevan a cabo entre dos y seis veces al año y fueron los encargados de dar a cada residente de las viviendas seleccionadas, una explicación completa, en su lengua, relacionada con el trabajo que se realizaría. Dicha esta explicación señalaba la relevancia y el objetivo del estudio. Sólo después de recibir el consentimiento verbal de los dueños de las viviendas se inició el estudio.

No se solicitó la aprobación del Comité de Bioética del Instituto Conmemorativo Gorgas, porque no se obtuvo ninguna información personal de los responsables de las viviendas. La aplicación de la nebulización fue realizada con la colaboración y participación del personal de control de vectores de la Región de Salud de Kuna Yala, quienes se ocupan de realizar los trabajos antivectoriales de rutina en las comunidades, siguiendo las directrices del Programa Nacional de Control de Aedes del Ministerio de Salud de Panamá.

## Resultados

### Bioensayos de sensibilidad con mosquitos adultos

**Figura 3.**
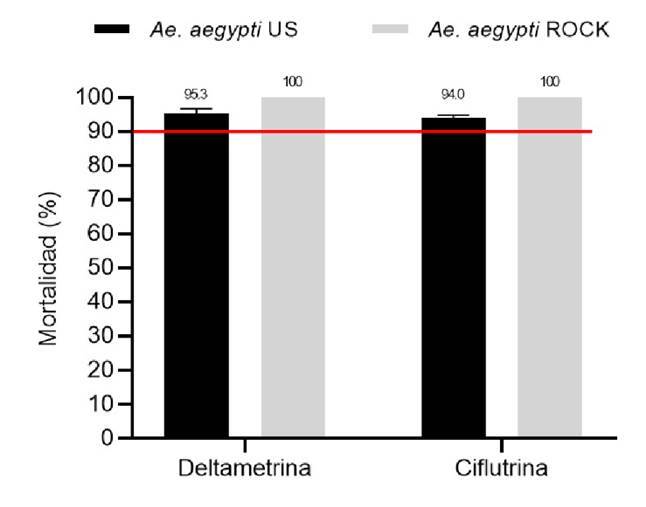
Porcentaje de mortalidad en bioensayos de sensibilidad con adultos de *Aedes aegypti* de Ustupo y de la cepa Rockefeller. La línea roja marca el umbral del 90 %, por debajo de este valor se considera que la población es resistente a los insecticidas. Los porcentajes de mortalidad están expresados como media geométrica más o menos el intervalo de confianza del 95 %.

Según los resultados obtenidos en los bioensayos de sensibilidad, la cepa *Ae. aegypti* Ustupo mostró una posible resistencia a la deltametrina con 95,3 % de mortalidad promedio y a la ciflutrina con 94 %. Los resultados de los bioensayos de sensibilidad se presentan en la figura 3 y en el cuadro 1.

**Cuadro 1.** Estado de la resistencia a insecticidas piretroides de adultos de *Aedes aegypti* de Ustupo, Comarca Kuna Yala, Panamá

Insecticida	Mosquitos (n)	Efecto knock-down 1 h expuestos después de la exposición n (%)	Mortalidad 24 h después de la exposición n (%)*	Sobrevivientes 24 h después de la exposición n (%)	Estado de la sensibilidad
Deltametrina	400	341 (85,3)	381 95,3 (93,1-97,4)	2 (0,5)	Posible resistencia
Ciflutrina	400	329 (82,3)	376 94,0 (91,6-96,4)	4(1,0)	Posible resistencia

* Los porcentajes de mortalidad están expresados como media geométrica ± el intervalo de confianza del 95 %.

### Pruebas de eficacia biológica con insecticidas piretroides deltametrina y ciflutrina

Durante la evaluación de los insecticidas piretroides, aplicados mediante nebulización térmica de volumen ultrabajo, la velocidad del viento no superó los 10 km/h y su dirección fue de norte a sur; la variación de la temperatura se mantuvo entre los 25 y los 31 °C y la humedad relativa osciló entre el 70 y el 80 %.

**Figura 4.**
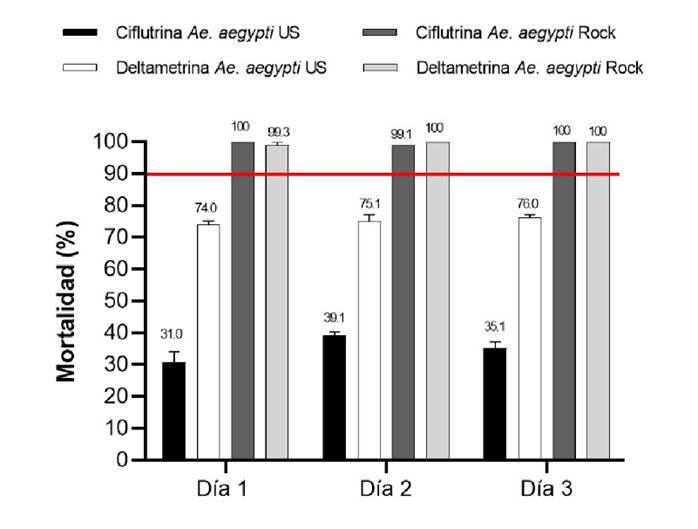
Porcentaje de mortalidad en el intradomicilio de *Aedes aegypti* de Ustupo y de la cepa Rockefeller, 24 horas después de su exposición a piretroides con nebulización térmica de volumen ultrabajo. La línea roja marca el umbral del 90 %, por debajo de este valor se considera que la población es resistente a los insecticidas. Los porcentajes de mortalidad están expresados como media geométrica más o menos el intervalo de confianza del 95 %.

**Figura 5.**
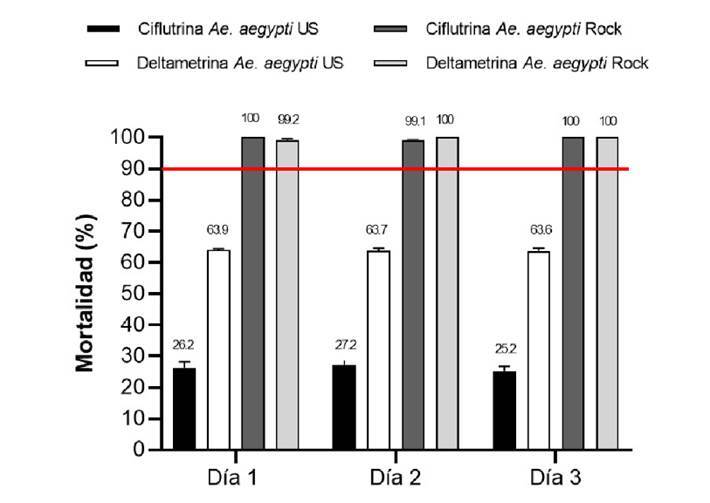
Porcentaje de mortalidad en el peridomicilio de *Aedes aegypti* de Ustupo y de la cepa Rockefeller, 24 horas después de su exposición a piretroides con nebulización térmica de volumen ultrabajo. La línea roja muestra el umbral del 90 %, por debajo de este valor se considera que la población es resistente a los insecticidas. Los porcentajes de mortalidad están expresados como media geométrica más o menos el intervalo de confianza del 95 %.

Los resultados del experimento se reportaron en términos de porcentaje de mortalidad para la cepa *Ae. aegypti* Ustupo y para los controles con la cepa sensible *Ae. aegypti* Rockefeller. El rango del porcentaje de mortalidad registrado en las pruebas de eficacia biológica en el intradomicilio para la cepa *Ae. aegypti* Ustupo con deltametrina en los tres días de aplicación fue entre el 74 y el 76 % de mortalidad y para ciflutrina entre el 31 y el 39,1 % (figura 4 y cuadro 2. El rango del porcentaje de mortalidad en el peridomicilio para la *Ae. aegypti* Ustupo con deltametrina en los tres días de aplicación estuvo entre el 63,6 y el 63,9 % y para ciflutrina entre el 25,2 y el 27,1 %. El rango del porcentaje de mortalidad en los controles con la cepa *Ae. aegypti* Rockefeller estuvo entre el 99,2 y el 100 %. Estos resultados muestran una baja eficacia biológica de la ciflutrina, y más baja para la deltametrina, contra mosquitos de la cepa *Ae. aegypti* Ustupo en comparación con la cepa control (Rockefeller), cuando son aplicadas con nebulización térmica de volumen ultrabajo (figura 5 y cuadro 3).

**Cuadro 2.** Porcentaje de mortalidad registrada para *Aedes aegypti* en jaulas colocadas en el intradomicilio durante la nebulización con insecticidas piretroides en Ustupo, Kuna Yala, Panamá

Insecticida	Concentración	Replica n	Jaulas n	Mosquitos expuestos n	Efecto knock Mortalidad 1 h down 1 h (%)		Mosquitos muertos 24 horas n	Mortalidad 24 horas (%)
Deltametrina	0,54%	2	30	450	281	62,4	333	74,0
		2	30	450	286	64,0	338	75,1
		2	30	450	275	61,1	342	76,0
Ciflutrina	0,12%	2	30	450	125	27,8	139	31,0
		2	30	450	131	29,1	176	39,1
		2	30	450	128	28,4	158	35,1

**Cuadro 3:** Porcentaje de mortalidad registrada de *Aedes aegypti* en jaulas colocadas en el peridomicilio durante la nebulización con insecticidas piretroides en Ustupo, Kuna Yala, Panamá

Insecticida	Concentración	Replica n	Jaulas n	Mosquitos expuestos n	Efecto knock Mortalidad 1 h down 1 h (%)		Mosquitos muertos 24 horas n	Mortalidad 24 horas (%)
Deltametrina	0,54%	2	30	449	256	57,0	286	63,7
		2	30	449	261	58,1	287	63,9
		2	30	448	253	56,4	285	63,6
Ciflutrina	0,12%	2	30	450	103	23,0	118	26,2
		2	30	449	99	22,0	122	27,2
		2	30	449	105	23,4	113	25,2

El cuadro 4 muestra el cociente de probabilidades de mortalidad de los mosquitos después de la exposición a ciflutrina y deltametrina. Con este modelo, se ajustaron los datos (DE = 42,636; gl = 43, p = 0,487). El nivel de referencia es el control de mosquitos Rockefeller en el ambiente domiciliario, una hora después de la exposición al insecticida. Los mosquitos en el peridomicilio tenían 32 % menos de probabilidades de morir que los que estaban dentro de los domicilios. Mientras tanto, la mortalidad de los mosquitos aumentó en casi 21 y 102 veces, cuando se expusieron a ciflutrina y deltametrina respectivamente. Independientemente del insecticida y el lugar de aplicación, 24 horas después de la aplicación del insecticida, los mosquitos tenían casi 14 veces más probabilidades morir.

**Cuadro 4.** Proporción de las probabilidades de mortalidad de individuos *Aedes aegypti* de Ustupo, después de ser expuestos a los insecticidas piretroides ciflutrina y deltametrina

Variable	Proporción de probabilidad	Estimación	Error estándar	Valor z Pr(>|z|)
Control intradomicilio 1 h	-	-6,264	0,166	37,67 < 2x10-^16^*
Peridomicilio	0,681	-0,384	0,050	7,62 < 2x10-^14^*
Ciflutrina	20,640	3,027	0,161	-18,83 <2x10-^16^*
Deltametrina	102,427	4,629	0,161	-28,84 <2x10-^16^*
24 h	13,890	2,631	0,060	-44,07 < 2x10-^16^*

* Estadísticamente significativo (P<0,005); valor z: coeficiente de regresión dividido entre el error estándar; Pr(>|z|): p valor asociado al valor z

## Discusión

### Bioensayos de sensibilidad

La deltametrina y la ciflutrina han sido utilizadas por el Programa Nacional de Control de Aedes desde 1996 contra las poblaciones de *Ae. aegypti* mediante aplicaciones de volumen ultrabajo con equipo pesado, portátil en frío y térmico. A pesar del uso de estos insecticidas desde hace 25 años, son pocos los trabajos que se han hecho para determinar la eficacia biológica y la sensibilidad de estos insecticidas piretroides en las poblaciones de Aedes de Panamá.

En la actualidad, los insecticidas piretroides siguen siendo los más utilizados, por lo que la resistencia de mosquitos vectores de arbovirus, altamente competentes como *Ae. aegypti*, está muy extendida ^42^. También se ha confirmado la resistencia de *Ae*. albopictus a estos compuestos ^43^.

Hasta 1962 se había logrado en las Américas, por medio del programa de erradicación, una efectiva lucha contra las poblaciones de *Ae. aegypti*, mediante la aplicación de dicloro-difenil-tricloroetano (DDT) ^44^. Sin embargo, el fracaso de las medidas implementadas y su seguimiento contribuyó a la repoblación del vector y la generación de poblaciones de mosquitos resistentes ^45^. En un gran número de países de las Américas, se ha estudiado la resistencia de *Ae. aegypti* a los insecticidas comúnmente utilizados para el control vectorial ^22^, como parte de las estrategias para evaluar la eficacia de los programas implementados y generar estrategias adicionales de control.

Los resultados de este estudio muestran por primera vez que la cepa de *Ae. aegypti* Ustupo presenta una posible resistencia a los adulticidas deltametrina y ciflutrina. La posible resistencia obervada en los bioensayos estandarizados por la OMS puede deberse a las aplicaciones continuas de estos insecticidas piretroides por parte del Programa Nacional de Control de Aedes. Generalmente las aplicaciones de estos insecticidas contra *Ae. aegypti* se hace a nivel de perifocos, alrededor de los casos reportados, en áreas delimitadas donde se registran los brotes de las enfermedades arbovirales y en áreas con altos índices de infestación.

Es importante indicar que la cobertura de aplicación de insecticidas no se realiza a gran escala y esto hace que las poblaciones de mosquitos de *Ae. aegypti* no estén expuestas continuamente a los insecticidas aplicados. Es posible que, por esta razón, la cepa *Ae. aegypti* Ustupo no presente una resistencia confirmada o establecida. La comunidad de Ustupo está ubicada en una isla, regiones que se consideran menos expuestas al uso excesivo de insecticidas o agroquímicos ^46^.

En consideración a lo observado en las pruebas biológicas de campo, que registraron poca eficacia biológica de los insecticidas deltametrina y ciflutrina, versus los bioensayos de sensibilidad que registraron una posible resistencia, cabe mencionar que la posible resistencia encontrada para deltrametrina y ciflutrina registró porcentajes de mortalidad (95,3 y 94 %, respectivamente) que pueden considerarse eficaces para el control de las poblaciones de *Aedes* spp. Sin embargo, debe mantenerse una vigilancia del comportamiento de dicha resistencia para que no se intensifique.

También debe considerarse que las diferencias observadas pueden deberse a las bajas concentraciones utilizadas de deltametrina (0,068 %) y ciflutrina de (0,167 %) para las nebulizaciones térmicas de volumen ultrabajo y, por ello, no se obtuvieron porcentajes de mortalidad más altos en las poblaciones de *Ae. aegypti* evaluadas.

Las concentraciones operativas probadas tienen más de 25 años de uso y no han sido evaluadas en el transcurso del tiempo. Por esta razón, el control de calidad de las aplicaciones de volumen ultrabajo y las pruebas de resistencia a los insecticidas deben ser un componente integrado a los programas nacionales de control de vectores ^47^.

Por otra parte, con este estudio se ha podido establecer que las poblaciones de *Ae. aegypti* de las zonas urbanas y semiurbanas muestran una resistencia, moderada a muy alta, a los insecticidas piretroides permetrina y deltametrina, y con una prevalencia mucho menor, también se ha detectado resistencia en sitios rurales ^48^.

La comarca de Kuna Yala es una de las principales regiones endémicas de malaria en el país, incluyendo Ustupo. El Programa Nacional de Malaria utiliza como una de las intervenciones, durante el registro de casos o brotes, las aplicaciones de deltametrina o ciflutrina con equipo termonebulizador de volumen ultrabajo, portátil, igual a las realizadas por el Programa Nacional de Control de Aedes, de manera que etas continuas aplicaciones pueden llegar a ejercer una presión selectiva contra las poblaciones de *Ae. aegypti*.

Varios trabajos indican que el uso intensivo de insecticidas es una fuerte presión de selección para los mosquitos y puede favorecer el aumento de las frecuencias alélicas asociadas con resistencia en poblaciones naturales. La resistencia ofrece una gran ventaja selectiva en entornos bajo presión de selección de insecticidas y, como consecuencia, los alelos resistentes a los insecticidas generalmente aumentan su frecuencia muy rápidamente en poblaciones de mosquitos naturales expuestas a aplicación intensa de insecticidas ^49^^,^^50^. En otro estudio se reportó que las nebulizaciones realizadas por los programas de malaria también pudieron haber afectado el estado de la resistencia de *Ae. aegypti* a insecticidas piretroides en las tres localidades estudiadas y que el uso de insecticidas domésticos también puede constituir una fuente importante de selección ^51^^,^^52^.

Es importante considerar los antecedentes genéticos de *Ae. aegypti* de diferentes lugares ya que podrían presentar distintos perfiles y mecanismos de resistencia a los insecticidas, según la región geográfica. Esta posible multiplicidad de situaciones justifica la importancia de monitorear la dinámica de resistencia a insecticidas de las poblaciones naturales de vectores, ya sea que estén expuestas a la presión de un determinado insecticida o para su intervención en el campo ^53^.

Considerando los resultados obtenidos en este estudio, es muy importante que el Programa Nacional de Control de Aedes desarrolle un programa que regularmente haga evaluaciones de la sensibilidad y la eficacia biológica de los insecticidas aplicados con nebulizaciones de volumen ultrabajo, pero en condiciones de campo para determinar el estado de la resistencia de las poblaciones de *Ae. aegypti*. Estos trabajos son de crítica importancia para los programas de control de vectores.

El conocimiento del nivel de sensibilidad y la eficacia biológica de los insecticidas aplicados en las intervenciones antivectoriales es esencial, especialmente en aquellas que buscan una reducción rápida de las densidades de las poblaciones de Ae. aegypti y la intensidad o el riesgo de transmisión de virus como CHIKV, DENV y ZIKV durante brotes epidémicos. Uno de los grandes problemas que limita la vigilancia y el control eficaz y efectivo de las poblaciones de *Ae. aegypti*, y en consecuencia la transmisión de las arbovirosis, es la pérdida de la capacidad de respuesta para prevenir y controlar estas enfermedades por parte de los programas sanitarios nacionales, lo cual constituye otro determinante de la expansión de esta enfermedad ^54^^-^^56^. Basado en estos hechos, en general los programas sanitarios y los sistemas de vigilancia son reactivos al reporte de actividades relacionadas con enfermedades como el dengue, por ende, el control es tardío y muy poco efectivo ^57^.

### Eficacia biológica de los insecticidas piretroides contra Aedes aegypti

Los estudios sobre la eficacia biológica de las aplicaciones de insecticidas con nebulizadores de volumen ultrabajo varían sustancialmente ^58^^,^^59^, ya que hay múltiples factores involucrados. Por ejemplo, cuando se aplican insecticidas con el equipo montado en un vehículo, la disponibilidad, las condiciones de las calles, la presencia y la altura de las paredes o muros, la ubicación de las casas dentro de los lotes y la presencia y el estado de las puertas y ventanas (abiertas o cerradas), todos influyen en la eficacia. Cuando se utiliza un equipo manual o portátil para este tipo de nebulizaciones, la eficacia puede variar en función del tiempo de aplicación en una casa, la minuciosidad de la aplicación y la presencia de sitios de reposo del mosquito inaplicables. Se sabe que *Ae. aegypti* reposa en lugares apartados e inalcanzables, como dentro de los armarios y debajo de las camas ^60^^,^^61^. Los factores relacionados con los insecticidas (tipo, dosis, tamaño de las gotas) y los factores ambientales (temperatura, dirección y velocidad del viento) también influyen en la eficacia de las aplicaciones ^58^^,^^61^.

Ustupo es una pequeña isla donde no existe ningún tipo de vehículo al igual que en todas las islas de la comarca de Kuna Yala. Tiene una sola calle principal y numerosas veredas, situación que hace imposible utilizar equipo pesado para la aplicación de nebulizaciones de volumen ultrabajo y en frío. Lo anterior, considerando que este tipo de aplicación no logra realizar una cobertura completa en sitios donde las calles son muy estrechas o no existen.

En los estudios realizados con equipo pesado de volumen ultrabajo se menciona que la nube del insecticida no logra penetrar en los sitios que sirven de refugios a *Ae. aegypti*, particularmente en zonas urbanas, donde los muros, edificios y otras estructuras obstruyen la deriva de las partículas de insecticidas. Asimismo, la nube de insecticida no logra penetrar eficazmente dentro de las viviendas y sitios que sirven de refugios para el *Ae. aegypti* en el peridomicilio ^61^.

Las aplicaciones de insecticidas a volumen ultrabajo con equipos portátiles, en frío o térmicas, son consideradas mucho más eficaces que las del equipo pesado, debido a que el personal técnico puede entrar a las viviendas y aplicar la nube de insecticidas, de forma más directa, en el interior de las viviendas y en diferentes sitios del peridomicilio que sirven de refugio a los mosquitos.

Este estudio presenta por primera vez evidencia de la baja eficacia biológica registrada de los insecticidas deltametrina y ciflutrina en *Ae. aegypti* Ustupo. Esta puede estar asociada a la limitada efectividad de las aplicaciones de piretroides con equipos de nebulizaciones de volumen ultrabajo y su capacidad para disminuir o controlar las poblaciones de mosquitos como medida para reducir la transmisión de los arbovirus. Los bioensayos con las jaulas de exposición demostraron que las dosis operativas utilizadas con el equipo de nebulización portátil, para deltametrina y ciflutrina, no lograron un porcentaje de mortalidad efectivo contra las muestras de *Ae. aegypti* Ustupo mientras que la totalidad de mosquitos de *Ae. aegypti* Rockefeller mueriron bajo las mismas condiciones experimentales.

Como se ya mencionó, estas dosis operativas para deltametrina y ciflutrina han sido utilizadas por 25 años y no se han evaluado periódicamente para determinar su eficacia biológica por parte del Programa Nacional de Control de Aedes, lo que puede constituir una falla en el control de las poblaciones de Aedes. La falta de efectividad de la deltametrina y la ciflutrina fue confirmada por los datos obtenidos a partir del análisis de las jaulas de exposición colocadas en el intradomicilio y en el peridomicilio. En estos bioensayos se obtuvo que, después de tres rondas diferentes de aplicación de los insecticidas piretroides, en días diferentes, no se tuvo un efecto acentuado en el número de hembras muertas de *Ae. aegypti*.

Teniendo en cuenta la mortalidad por encima del 90 % de los bioensayos de sensibilidad, que indicaba una posible resistencia a los insecticidas piretroides evaluados, y la baja eficacia biológica de las dosis operativas probadas, podemos asumir que el porcentaje de mortalidad registrado en los bioensayos de sensibilidad puede resultar eficaz para disminuir y controlar hasta cierto nivel las poblaciones de *Ae. aegypti*. No obstante, es necesario mantener una vigilancia estrecha del comportamiento de la resistencia para prevenir que se intensifique.

Además, se requiere que el Programa Nacional de Control de *Aedes* evalúe aplicar nuevas dosis o diferentes concentraciones operativas para ambos insecticidas, que puedan aplicarse en nebulizaciones de volumen ultrabajo, y que resulten más eficaces y efectivas para controlar las poblaciones de *Aedes*. Esto permitiría, al mismo tiempo, mantener una vigilancia y monitoreo de la eficacia biológica de las nuevas dosis operativas mediante investigaciones técnicas.

Por otra parte, la mortalidad total de las hembras de *Ae. aegypti* Rockefeller mantenidas en las jaulas de exposición, colocadas en el intradomicilio y el peridomicilio, demuestra que el insecticida pudo penetrar en los diferentes sitios de las casas. Sin embargo, estos resultados deben tomarse con precaución ya que, en condiciones naturales, parte de la población de mosquitos reposa en lugares confinados y protegidos (por ejemplo, debajo de las camas y muebles, dentro de armarios) donde es posible que el insecticida no siempre entre en contacto con los mosquitos.

A pesar de que la deltametrina registró una mayor eficacia biológica en comparación con la ciflutrina, este porcentaje de mortalidad puede considerarse bajo para lograr un impacto en la reducción de las poblaciones de *Ae. aegypti*. Se puede considerar entre un 80 y un 90 % de mortalidad como un porcentaje capaz de causar un impacto para lograr disminuir significativamente las poblaciones de mosquitos y prevenir la transmisión de los ZIKV, CHIKV y DENV. Esto debe ir acompañado por una buena cobertura espacial y frecuencia de aplicación.

*Aedes aegypti* es una especie endofílica que pasa gran parte de su tiempo en sitios protegidos, en el interior de las viviendas como, por ejemplo, entre la ropa en los armarios. En un estudio realizado en México se encontró que la mayoría (99 %) de las hembras de *Ae. aegypti* reposaban en dormitorios, salas, baños y cocinas, donde los individuos predominantemente (82 %) descansan a menos de 1,5 m del piso, y no necesariamente en las paredes, por lo cual el rociado residual intradomiciliario no es una opción adecuada para su control ^62^^,^^63^. Por el contrario, Ae. albopictus muestra una preferencia por sitios extradomiciarios ^64^.

En experimentos a pequeña escala, cuidadosamente controlados, se realizaron nebulizaciones de volumen ultrabajo con el equipo portátil en espacios interiores. Como resultado se obtuvo que a menudo las poblaciones de adultos de *Ae. aegypti* disminuían en más del 80 %. Las aplicaciones de aerosoles espaciales en interiores logran altos niveles de mortalidad de adultos a pesar del comportamiento de reposo de *Ae. aegypti*^65^^,^^66^. Sin embargo, las densidades poblacionales se recuperaban rápidamente ^60^ debido a la aparición de hembras nulíparas que migran desde el hábitat fuera de las áreas nebulizadas y hembras que sobrevivieron en casas donde se nebulizó con insecticidas ^67^.

En un trabajo similar con *Ae. aegypti* en Venezuela, la mortalidad fue superior al 90 % en mosquitos que estaban en jaula en sitios abiertos, pero fue cerca de cero en sitios del intradomicilio donde los mosquitos están frecuentemente en reposo ^68^. Por su parte, Andis *et al*. registraron una mortalidad del 90 % de mosquitos expuestos en jaulas y entre el 34 y el 67 % en mosquitos que se encontraban reposando sobre la vegetación ^69^.

El reporte de Bonds indica que hay una probabilidad 100 veces mayor de que una gotita aplicada con un equipo térmico portátil para aplicación de volumen ultrabajo entre en contacto con un mosquito colocado en una jaula versus una aplicación del mismo tipo, pero con equipo pesado. Esto se debe a que el personal técnico de vectores puede entrar a las viviendas con el equipo portátil y hacer la aplicación del insecticida en los diferentes sitios del intradomicilio y el peridomicilio, mientas que el equipo pesado, montado en un vehículo, realiza la aplicación del insecticida desde la calle y no penetra con gran efectividad en el intradomicilio ^70^.

En muchos países, la aplicación de insecticidas con equipo de nebulización de volumen ultrabajo, pesado y portátil, se considera el método primario de elección durante las emergencias de salud pública ^71^. La estrategia principal para el control del dengue consiste en controlar las poblaciones de los vectores mediante la nebulización con insecticidas como una intervención reactiva a la transmisión local en curso. Sin embargo, esta práctica rara vez se ha evaluado en términos de su impacto en la transmisión de dengue en situaciones de la vida real ^62^^,^^72^. Dicha evaluación se dificulta por las interacciones intrínsecamente complejas entre las poblaciones de mosquitos, la transmisión viral, la absorción de insecticidas en espacios estructurados y las condiciones ambientales variables en el tiempo ^70^.

En una revisión sobre la efectividad del control de vectores para el dengue ^73^, se concluyó que, aunque la nebulización espacial de volumen ultrabajo es la respuesta de salud pública estándar a un brote de dengue en todo el mundo, y que la OMS ^74^ recomienda para este fin, existe poca evidencia disponible que evalúe este método. En otro trabajo realizado, se indica que los ensayos de campo con aplicaciones de volumen ultrabajo no han demostrado un impacto significativo contra poblaciones de *Ae. aegypti* en ambientes urbanos y que no hay evidencia del impacto favorable de estas aplicaciones sobre la disminución de la transmisión viral ^68^.

Además, incluso si la aplicación de insecticidas con nebulizaciones de volumen ultrabajo lograra una reducción importante en las poblaciones de mosquitos, el efecto sería probablemente transitorio en la reducción de la transmisión viral por la recuperación rápida de las poblaciones ^72^.

Con el análisis realizado mediante el uso del modelo lineal binomial generalizado, se pudo confirmar que la cepa Ae. a*egypti* Ustupo fue significativamente más sensible a la interacción de la deltametrina aplicada en el intradomicilio que con la ciflutrina. Estudios que utilizaron este mismo método de análisis señalaron que cuando se monitorea la aparición de resistencia en una población, es importante mantener las variables constantes para hacer comparaciones sólidas entre las réplicas de la prueba e identificar cambios reales en los resultados a lo largo del tiempo ^75^^,^^76^. En un estudio previo con *Ae. aegypti*, en el que se realizó la prueba estándar de senesibilidad de la OMS, las hembras de mosquitos de diez días de emergidas fueron significativamente más sensibles a la deltametrina que las de tres a cinco días ^77^.

Reportes previos registraron que las densidades de *Ae. aegypti* disminuyen significativamente después del primer ciclo de rociado y luego fluctúan a niveles relativamente bajos durante los ciclos de rociado restantes. En tres intervenciones realizadas, las poblaciones adultas se recuperaron parcialmente dentro de las dos semanas posteriores al cese del rociado ^78^^,^^79^. Estudios realizados en Honduras y Costa Rica demostraron una reducción, aproximadamente, del 90 % de mosquitos adultos en la primera semana después de la nebulización, pero el efecto de la aplicación no fue significativo después de la semana seis a siete ^80^^,^^81^.

Un aspecto técnico para destacar es que, por lo general, en los sitios donde se hacen aplicaciones de insecticidas con equipo de nebulización de volumen ultrabajo, se desconoce o no se tienen datos sobre los niveles de resistencia a los insecticidas aplicados en la zona ^82^. Dado el fuerte efecto negativo que la resistencia a los insecticidas puede tener sobre la eficacia de las intervenciones ^83^, es crucial que las nebulizaciones de volumen ultrabajo y cualquier otra intervención con insecticidas se implementen dentro de un plan de manejo de la resistencia a estos insecticidas. La aplicación de emergencia exitosa durante los brotes requeriría mecanismos para la adquisición de insecticidas que sean rápidos y basados en el perfil de resistencia de las poblaciones locales de *Ae. aegypti*.

Con base en los resultados de este estudio, se puede concluir que el estado de la resistencia en las distintas regiones de importancia epidemiológica y entomológica representa un desafío que debe enfrentar el Programa Nacional de Control de Aedes para mantener el control de las poblaciones de *Ae. aegypti* con los insecticidas regularmente utilizados. Es necesario que el Programa Nacional de Control de *Aedes* establezca unos lineamientos de vigilancia y manejo de la resistencia para evaluar su distribución e intensidad. Esto con el propósito de garantizar la sostenibilidad de las intervenciones antivectoriales contra las poblaciones de *Ae. aegypti*.

## References

[1] Bhatt S, Gething PW, Brady OJ, Messina JP, Farlow AW, Moyes CL (2013). The global distribution and burden of dengue. Nature.

[2] Musso D, Cao-Lormeau VM, Gubler DJ (2015). Zika virus: following the path of dengue and chikungunya?. Lancet.

[3] Vorou R. (2016). Zika virus, vectors, reservoirs, amplifying hosts, and their potential to spread worldwide: what we know and what we should investigate urgently. Int J Infect Dis.

[4] Zanluca C, De Melo VC, Mosimann AL, Gl Dos Santos, Dos Santos CN, Luz K (2015). First report of autochthonous transmission of Zika virus in Brazil. Mem Inst Oswaldo Cruz.

[5] European Centre for Disease Prevention and Control (2015). Zika virus epidemic in the Americas: potential association with microcephaly and Guillain-Barré syndrome.

[6] Centers for Disease Control and Prevention (2017). Surveillance and control of *Aedes aegypti* and Aedes albopictus in the United States.

[7] Pan American Health Organization (2017). Zika cumulative cases.

[8] Organización Panamericana de la Salud (2022). Plataforma de información en salud para las Américas.

[9] Cardoso CW, Paploski I, Kikuti M, Rodrigues MS, Silva M, Campos GS (2015). Outbreak of exanthematous illness associated with Zika, Chikungunya, and dengue viruses, Salvador, Brazil. Emerg Infect Dis.

[10] Braks MAH, Honorio NA, Lounibos LP, Lourengo-de-Oliveira R, Juliano AS (2004). Interespecific competition between two invasive species of container mosquitoes, Aedes aegypti and Aedes albopictus (Díptera: Culicidae) in Brazil. Ann Entomol SocAm.

[11] Maciel-de-Freitas R, Marques WA, Peres RC, Cunha SP, de Oliveira RL (2007). Variation in Aedes aegypti (Díptera: Culicidae) container productivity in a slum and a suburban district in Rio de Janeiro during dry and wet seasons. Mem Inst Oswaldo Cruz.

[12] Bisset JA, Rodriguez MM, Cáceres L (2003). Levels of resistance to insecticides and their mechanisms in 2 strains of Aedes aegypti from Panama. Rev Cubana Med Trop.

[13] Wilson AL, Courtenay O, Kelly-Hope LA, Scott TW, Takken W, Torr SJ (2020). The importance of vector control for the control and elimination of vector-borne diseases. PLoS Negl Trop Dis.

[14] Sridhar S, Luedtke A, Langevin E, Zhu M, Bonaparte M, Machabert T (2018). Effect of dengue serostatus on dengue vaccine safety and efficacy. N Engl J Med.

[15] Marcombe S, Poupardin R, Darriet F, Reynaud S, Bonnet J, Strode C (2009). Exploring the molecular basis of insecticide resistance in the dengue vector Aedes aegypti: a case study in Martinique Island (French West Indies). BMC Genomics.

[16] Guzmán MG, Kouri GP, Bravo J, Soler M, Vazquez S, Morier L (1990). Dengue hemorrhagic fever in Cuba, 1981: a retrospective seroepidemiologic study. Am J Trop Med Hyg.

[17] LaMer VK, Hochberg S, Hodges K, Wilson I, Fales JA, Latta R (1947). The influence of the particle size of homogeneous insecticidal aerosols on the mortality of mosquitoes in confined atmospheres. J Colloid Sci.

[18] Lofgren CS. (1970). Ultralow volume applications of concentrated insecticides in medical and veterinary entomology. Ann Rev Entomol.

[19] Brown AWA (1986). Insecticide resistance in mosquitoes: a pragmatic review. J Am Mosq Control Assoc.

[20] Bisset JA, Rodriguez MM, Moya M, Ricardo Y, Montada D, Gato R (2011). Efectividad de formulaciones de insecticidas para el control de adultos de Aedes aegypti en La Habana, Cuba. Rev Cubana Med Trop.

[21] Rawlins SC (1998). Spatial distribution of insecticide resistance in Caribbean populations of Aedes aegypti and its significance. Rev Panam Salud Publica.

[22] Rodriguez MM, Bisset JA, Fernandez D (2007). Levels of insecticide resistance and resistance mechanisms in Aedes aegypti from some Latin American countries. J Am Mosq Control Assoc.

[23] Bisset JA, Rodriguez MM, San Martín JL, Romero JE, Montoya R (2009). Evaluación de la resistencia a insecticidas de una cepa de Aedes aegypti de El Salvador. Rev Panam Salud Publica.

[24] Cáceres L. (1999). La lucha antimalárica en Panamá. Primera edición.

[25] Ministerio de Salud de Panamá (2016). Informe técnico epidemiológico del brote por infección del virus zika en la Comarca de Kuna Yala.

[26] Ministerio de Salud de Panamá Decreto Ejecutivo No. 1218 del 7 de diciembre de 2015.

[27] Guzmán HM, Guevara C, Castillo A (2003). Natural disturbances and mining of Panamanian coral reefs by indigenous people. Conserv Biol.

[28] Calzada JE, Marquez R, Rigg C, Victoria C, De La Cruz M, Chaves LF (2015). Characterization of a recent malaria outbreak in the autonomous indigenous region of Guna Yala, Panama. Malar J.

[29] Contraloria General de la República de Panamá, Instituto Nacional de Estadística y Censo Boletín No. 15. Estimación y proyecciones de la población en la república, provincias, comarcas indígenas por distrito, según sexo y edad; 2010-2020.

[30] Martínez Mauri M. (2011). La autonomía indígena en Panamá: la experiencia del pueblo kuna (siglos XVI-XXI).

[31] Instituto Metereológico Hidrológico de Panamá Datos históricos.

[32] Clark-Gil S, Darsie RF (1983). The mosquitoes of Guatemala. Mosq Sist.

[33] Cáceres L, Rovira J, García A, Torres R, De La Cruz M (2013). Determinación de la sensibilidad a insecticidas OP, CAy PY en poblaciones de Aedes aegypti Linneaus, 1762 (Díptera: Culicidae) de Panamá. Biomédica.

[34] Reiter P, Nathan MB, World Health Organization (2001). Guidelines for assessing the efficacy of insecticidal space sprays for control of the dengue vector *Aedes aegypti*.

[35] Ministerio de Salud de Panamá (2014). Guías para el abordaje integral del dengue en Panamá.

[36] Griffith M, Rovira J, Torres R, Calzada J, Victoria C, Cáceres L (2015). Conocimientos, actitudes y prácticas sobre la malaria en la población indígena Kuna de la comarca de Madungandí, Panamá, 2012. Biomédica.

[37] Najera JA, Kouznetsov RL, Delacollette C (1998). Malaria epidemics. Detection and control, forecasting and prevention.

[38] World Health Organization (1981). Instructions for determining the susceptibility or resistance of adult mosquitoes to organochlorine, organophosphorous and carbamato insecticides.

[39] World Health Organization (2016). Monitoring and managing insecticide resistance in Aedes mosquito populations Interim guidance for entomologists.

[40] Boubidi SC, Roiz D, Rossignol M, Chandre F, Benoit R, Raselli M (2016). Efficacy of ULV and thermal aerosols of deltamethrin for control of Aedes albopictus in Nice, France. Parasit Vectors.

[41] Abbott WS. (1925). A method for computing the effectiveness of an insecticide. J Economic Entomol.

[42] Amelia-Yap ZH, Chen CD, Sofian-Azirun M, Low VL (2018). Pyrethroid resistance in the dengue vector Aedes aegypti in Southeast Asia: present situation and prospects for management. Parasit Vectors.

[43] Smith LB, Kasai S, Scott JG (2016). Pyrethroid resistance in Aedes aegypti and Aedes albopictus: important mosquito vectors of human diseases. Pestic Biochem Physiol.

[44] Brathwaite DO, San Martín JL, Montoya RH, del Diego J, Zambrano B, Dayan GH (2012). The history of dengue outbreaks in the Americas. Am J Trop Med Hyg.

[45] Kraemer MU, Sinka ME, Duda KA, Mylne AQ, Shearer FM, Barker CM (2015). The global distribution of the arbovirus vectors Aedes aegypti and Ae. albopictus. Elite.

[46] Garcia GA, Hoffmann AA, Maciel-de-Freitas R, Villela DAM (2020). Aedes aegypti insecticide resistance underlies the success (and failure) of Wolbachia population replacement. Sci Rep.

[47] World Health Organization (2009). Dengue: guidelines for diagnosis, treatment, prevention, and control: new edition.

[48] García GP, Flores AE, Fernández-Salas I, Saavedra-Rodríguez K, Reyes-Solis G, Lozano- Fuentes S (2009). Recent rapid rise of a permethrin knock down resistance allele in Aedes aegypti in México. Píos Negl Trop Dis.

[49] Lima EP, Paiva MHS, Araújo AP, Silva EVG, Silva UM, de Oliveira LN (2011). Insecticide resistance in Aedes aegypti populations from Ceará, Brazil. Parasit Vectors.

[50] Gray L, Florez SD, Barreiro AM, Vadillo-Sánchez J, González-Olvera G, Lenhart A (2018). Experimental evaluation of the impact of household aerosolized insecticides on pyrethroid resistant Aedes aegypti. Sci Rep.

[51] Macoris ML, Martins AJ, Andrighetti MT, Lima JB, Valle D (2018). Pyrethroid resistance persists after ten years without usage against Aedes aegypñ in governmental campaigns: Lessons from Sao Paulo State, Brazil. PLoS Negl Trop Dis.

[52] San Martín JL, Brathwaite O, Zambrano B, Solórzano JO, Bouckenooghe A, Dayan GH (2010). The epidemiology of dengue in the Americas over the last three decades: A worrisome reality. Am J Trop Med Hyg.

[53] Garcia GA, David MR, Martins AJ, Maciel-de-Freitas R, Linss JG, Araújo SC (2018). The impact of insecticide applications on the dynamics of resistance: The case of four Aedes aegypti populations from different Brazilian regions. PLoS Negl Trop Dis.

[54] Mayer SV, Tesh RB, Vasilakis N (2017). The emergence of arthropod-borne viral diseases: A global prospective on dengue, chikungunya and zika fevers. Acta Trop.

[55] Gubler DJ. (2011). Dengue, urbanization and globalization: The Unholy Trinity of the 21st Century. Trop Med Health.

[56] Corbel V, Achee NL, Chandre F, Coulibaly MB, Dusfour I, Fonseca DM (2016). Tracking insecticide resistance in mosquito vectors of arboviruses: The Worldwide Insecticide Resistance Network (WIN). PLoS Negl Trop Dis.

[57] Khormi HM, Kumar L (2011). Modeling dengue fever risk based on socioeconomic parameters, nationality and age groups: GIS and remote sensing-based case study. Sci Total Environ.

[58] Gratz NG (1991). Emergency control of Aedes aegypti as a disease vector in urban areas. J Am Mosq Control Assoc.

[59] Gubler DJ (1989). Aedes aegypti and Aedes aegypti-borne disease control in the 1990s: top down or bottom up. Charles Franklin Craig Lecture. Am J Trop Med Hyg.

[60] Koenraadt CJ, Aldstadt J, Kijchalao U, Kengluecha A, Jones JW, Scott TW (2007). Spatial and temporal patterns in the recovery of Aedes aegypti (Díptera: Culicidae) populations after insecticide treatment. J Med Entomol.

[61] Perich MJ, Davila G, Turner A, Garcia A, Nelson M (2000). Behavior of resting Aedes aegypti (Culicidae: Díptera) and its relation to ultra-low volume adulticide efficacy in Panama City, Panama. J Med Entomol.

[62] Esu E, Lenhart A, Smith L, Horstick O (2010). Effectiveness of peridomestic space spraying with insecticide on dengue transmission; systematic review. Trop Med Int Health.

[63] Dzul-Manzanilla F, Ibarra-Lopez J, Bibiano Marin W, Martini-Jaimes A, Leyva JT, Correa- Morales F (2017). Indoor resting behavior of Aedes aegypti (Díptera: Culicidae) in Acapulco, Mexico. J Med Entomol.

[64] Boubidi SC, Roiz D, Rossignol M, Chandre F, Benoit R, Raselli M (2016). Efficacy of ULV and thermal aerosols of deltamethrin for control of Aedes albopictus in Nice, France. Parasit Vectors.

[65] Perich MJ, Sherman C, Burge R, Gill E, Quintana M, Wirtz RA (2001). Evaluation of the efficacy of lambda-cyhalothrin applied as ultra-low volume and thermal fog for emergency control of Aedes aegypti in Honduras. J Am Mosq Control Assoc.

[66] Perich MJ, Rocha NO, Castro AL, Alfaro AW, Platt KB, Solano T (2003). Evaluation of the efficacy of lambda-cyhalothrin applied by three spray application methods for emergency control of Aedes aegypti in Costa Rica. J Am Mosq Control Assoc.

[67] Reiter P., Gubler D, Ooi E, Vasudevan S, Farrar J (2014). Dengue and dengue hemorrhagic fever.

[68] Bengoa M, Eritja R, Lucientes J (2014). Ground ultra-low volume adulticiding field trials using pyrethroids against Aedes albopictus in the Baix Llobregat region, Spain. J Am Mosq Control Assoc.

[69] Andis MD, Sackett SR, Carroll MK, Bordes ES (1987). Strategies for the emergency control of arboviral epidemics in New Orleans. J Am Mosq Control Assoc.

[70] Bonds JAS (2012). Ultra-low-volume space sprays in mosquito control: a critical review. Med Vet Entomol.

[71] Bowman LR, Donegan S, McCall PJ (2016). Is dengue vector control deficient in effectiveness or evidence? Systematic review and meta-analysis. PLoS Negl Trop Dis.

[72] Gunning CE, Okamoto KW, Astete H, Vasquez GM, Erhardt E, Del Aguila C (2018). Efficacy of Aedes aegypti control by indoor Ultra Low Volume (ULV) insecticide spraying in Iquitos, Peru. PLoS Negl Trop Dis.

[73] World Health Organization (2011). Regional Office for South-East Asia. Comprehensive guidelines for prevention and control of dengue and dengue haemorrhagic fever. Revised and expanded edition.

[74] Newton EA, Reiter PA (1992). A model of the transmission of dengue fever with an evaluation of the impact of ultra-low volume (ULV) insecticide applications on dengue epidemics. Am J Trop Med Hyg.

[75] Rajatileka S, Burhani J, Ranson H (2011). Mosquito age and susceptibility to insecticides. Trans R Soc Trop Med Hyg.

[76] Machani MG, Ochomo E, Sang D, Bonizzoni M, Zhou G, Githeko AK (2019). Influence of blood meal and age of mosquitoes on susceptibility to pyrethroids in Anopheles gambiae from Western Kenya. Malar J.

[77] Al Nazawi AM, Aqili J, Alzahrani M, McCall PJ, Weetman D (2017). Combined target site (kdr) mutations play a primary role in highly pyrethroid resistant phenotypes of Aedes aegypti from Saudi Arabia. Parasit Vectors.

[78] Horstick O, Runge-Ranzinger S, Nathan MB, Kroeger A (2010). Dengue vector-control services: how do they work? A systematic literature review and country case studies. Trans R Soc Trop Med Hyg.

[79] Perich MJ, Sherman C, Burge R, Gill E, Quintana M, Wirtz RA (2001). Evaluation of the efficacy of lambda-cyhalothrin applied as ultra-low volume and thermal fog for emergency control of Aedes aegypti in Honduras. J Am Mosq Control Assoc.

[80] Perich MJ, Rocha N O, Castro A L, Alfaro A W, Platt KB, Solano T (2003). Evaluation of the efficacy of lambda-cyhalothrin applied by three spray application methods for emergency control of Aedes aegypti in Costa Rica. J Am Mosq Control Assoc.

[81] Hetzel MW, Pulford J, Ura Y, Jamea-Maiasa S, Tandrapah A, Tarongka N (2017). Insecticide- treated nets and malaria prevalence, Papua New Guinea, 2008-2014. Bull World Health Organ.

[82] Kuri-Morales P, Correa-Morales F, González-Acosta C, Moreno-García M, Santos-Luna R, Román-Pérez S (2018). Insecticide susceptibility status in Mexican populations of Stegomyia aegypti (= Aedes aegypti): a nationwide assessment. Med Vet Entomol.

[83] Vazquez-Prokopec GM, Medina-Barreiro A, Che-Mendoza A, Dzul-Manzanilla F, Correa- Morales F, Guillermo-May G (2017). Deltamethrin resistance in Aedes aegypti results in treatment failure in Merida, Mexico. PLoS Negl Trop Dis.

